# A Repair Method for Missing Traffic Data Based on FCM, Optimized by the Twice Grid Optimization and Sparrow Search Algorithms

**DOI:** 10.3390/s22114304

**Published:** 2022-06-06

**Authors:** Pengcheng Li, Baotian Dong, Sixian Li, Rusi Chu

**Affiliations:** 1 School of Traffic and Transportation, Beijing Jiaotong University, Beijing 100044, China; bjtulpc@163.com (P.L.); 20114056@bjtu.edu.cn (S.L.); 2 Henan Communications Planning & Design Institute Co., Ltd., Zhengzhou 450046, China; rusichu@126.com

**Keywords:** missing sensor data, fuzzy C-means, data repair mode, twice grid optimization algorithm, sparrow search algorithm

## Abstract

Complete traffic sensor data is a significant prerequisite for analyzing the changing rules of traffic flow and formulating traffic control strategies. Nevertheless, the missing traffic data are common in practice. In this study, an improved Fuzzy C-Means algorithm is proposed to repair missing traffic data, and three different repair modes are established according to the correlation of time, space, and attribute value of traffic flow. First, a Twice Grid Optimization (TGO) algorithm is proposed to provide a reliable initial clustering center for the FCM algorithm. Then the Sparrow Search Algorithm (SSA) is used to optimize the fuzzy weighting index m and classification number k of the FCM algorithm. Finally, an experimental test of the traffic sensor data in Shunyi District, Beijing, is employed to verify the effectiveness of the TGO-SSA-FCM. Experimental results showed that the improved algorithm had a better performance than some traditional algorithms, and different data repair modes should be selected under different miss rate conditions.

## 1. Introduction

Various traffic sensors have been widely installed in the urban road network to obtain traffic data such as traffic flow, driving speed, time occupancy, following percentage, headway, and so on, which lays a solid foundation for traffic managers to analyze traffic flow change rules, evaluate traffic conditions, and formulate traffic control measures [1,2].

However, missing data are very common in reality. They are easily affected by some conditions, such as changeable weather, interruption of communication and power, equipment hardware failure, etc. It will become an obstacle to traffic data analysis, as some widely used data mining algorithms, such as support vector machine [3], neural network [4], and ensemble learning [5], need the support of complete data sets in their specific applications. Consequently, how to repair the missing traffic data has become one of the hot issues in the current research.

Currently, traffic missing data is mainly addressed in traffic flow. In terms of repair, it is mainly divided into the statistics-based method and machine learning-based method [6]. The most common method based on statistics is the historical mean method [7], which uses the historical data collected by the same traffic sensor to calculate the average value to complete the missing data. The historical mean algorithm is a simple completion method, but there is a deviation between the mean and the actual traffic. When there are multiple missing values, the error between historical mean and actual traffic will continue to accumulate, resulting in an increasingly large deviation of the overall data, which seriously affects the accuracy of data restoration. There are also some regression algorithms with the same problems, such as Linear Fitting [8], Auto Regression Integrated Moving Average (ARIMA) [9,10], and so on. The less existing data there is, the harder it is to fit a reliable regression curve through the existing data. In addition, statistics-based methods include Functional Principal Component Analysis (FPCA) [11], Probabilistic Principal Component Analysis (PPCA), and so forth. Related studies showed that this type of algorithm based on principal component analysis had higher accuracy than the historical mean algorithm [12]. Traffic flow data should be interpreted in light of a probability distribution law. However, in reality, the traffic flow data are variable.

The learning-based method is to extract the complete samples from the incomplete data set as the training set and establish a model to predict the missing value. Compared with the statistics-based method, it does not need to assume the distribution law of traffic flow in advance; the model structure is relatively simple.

Existing machine learning methods mostly use the relevant characteristics of traffic flow (including time correlation [13], spatial correlation [14], attribute value correlation [15]) to compose data with high correlation into a data matrix, and then use various machine learning models to predict the missing values. To compare and analyze the above three characteristics, we set up three data repair modes, which are named Repair mode based on Time correlation (TR), Repair mode based on Spatial correlation (SR), and Repair mode based on Attribute value correlation (AR).

The rest of this paper is organized as follows: Section 2 introduces the related research of different methods to repair missing traffic data. Section 3 introduces the theory of three restoration modes and specific methods. Section 4 demonstrates an experimental test of measured data of traffic sensors in Beijing in 2018, and compared and analyzed the traffic flow data repair effect of six different experimental schemes. Section 5 summarizes the work of the whole paper and looks ahead to the direction of future research.

## 2. Related Work

Most of the existing missing data repair modes are TR or SR. In the TR mode, Tang et al. [13] and Huang et al. [16] distinguished the traffic flow data of the same traffic sensor on working days and non-working days in the same week, forming data matrices. Moreover, they proposed a traffic data estimation method based on Fuzzy C-Means (FCM) algorithm. The Genetic Algorithm (GA) was used to optimize the fuzzy weighted index m and clustering number K of FCM. Shang et al. [15] proposed an estimation to jointly optimize FCM parameters based on Particle Swarm Optimization (PSO) and Support Vector Regression (SVR). The actual traffic flow data of the Shanghai expressway were selected to verify the estimation effect. Luo et al. [17] formed a matrix with time-dependent data. They proposed a repair method based on Improved Low-Rank Matrix Decomposition (ILRMD), which verified the effectiveness by the measured traffic flow data of the Caltrans Performance Measurement System (PeMS). Han et al. [18] proposed a hierarchical probabilistic model to repair missing data. It comprised Bayesian tensor decomposition and a Dirichlet process mixture model, and the measured data of the same traffic sensor on Guangzhou road in one week were selected for verification.

In the SR mode, Henrickson et al. [19] collected volume data from two adjacent sensors on Interstate 5 in Washington State. The predictive mean matching multiple imputation method was adopted to repair the missing data. Shang et al. [20] proposed an optimal closed cut Optimum Closed Cut (OCC) method, which used the correlation of traffic data on closed cut intersecting roads to estimate the missing data but could only be predicted in hours. Zhang et al. [21] combined the traffic flow data of adjacent roads in the same period and used the algorithm of Integrated Bayesian Tensor Decomposition (IBTD) to repair missing data. Chen et al. [22] proposed a method of ensemble correlation-based low-rank matrix completion, which used the K-Nearest Neighbor (KNN) algorithm to screen the strong correlation samples and apply it to the traditional LRMC algorithm to obtain an accurate estimation of the missing values.

Previous studies had shown that better repair results could often be achieved by combining spatio-temporal traffic flow data into a data matrix, whether in TR mode or SR mode, although there were still some deficiencies in existing studies. For example, the research based on the FCM algorithm has not solved the problem of a random selection of the initial clustering center. The selected parameter optimization algorithms, such as the PSO algorithm, are easy to fall into local optimal, which will affect the final repair effect. In addition, only the flow data were selected when selecting the adjacent sensor data, and other attribute values such as speed, time occupancy, time-spent-following percent, headway, etc. Furthermore, there is a strong correlation between attributes [15,23]. More importantly, there will be outliers in individual attributes collected by traffic sensors. For example, traffic sensors based on infrared technology have a high recognition rate of vehicle types. However, it is easy to cause vehicle type recognition errors in bad weather, which will lead to errors in traffic flow because, in some countries, such as China, traffic flow is converted according to different vehicle type coefficients. Nevertheless, other attributes such as speed and time occupancy are relatively accurate and are not affected by the vehicle types.

Therefore, we proposed the AR mode, which assumed that abnormal traffic flow also belonged to missing data, and used correct attribute values such as speed and time occupancy to repair the missing data.

The FCM algorithm is a matrix-based clustering method that has been widely applied to the clustering problem of incomplete data sets [24] and achieved good results in traffic data clustering [25]. This study is based on the FCM algorithm for data repair. There are two main problems in the traditional FCM algorithm: (1) The initial clustering center is randomly selected, which will lead to different results of FCM each time. (2) The fuzzy weighted index m and classification number K of the model parameters must be selected manually, and the clustering results are sensitive to the above parameters [26]. Aiming at problem 1, we proposed a TGO (Twice Grid Optimization) algorithm, which divided the data matrix into grids and optimized twice according to the original distribution law of the data to provide a reliable and relatively fixed initial clustering center for FCM. Aiming at problem 2, we chose SSA (Sparrow Search Algorithm) to find the best combination of m and K. The SSA sparrow search algorithm is a new type of intelligent optimization algorithm proposed by Xue [27] in 2020 based on the predation law of sparrows. Compared with traditional swarm intelligent optimization algorithms such as GSA (Gravitational Search Algorithm), PSO, GWO (Gray Wolf Optimization algorithm), BA (Bat Algorithm), GOA (Grasshopper Optimization Algorithm), DA (Dragonfly Algorithm), etc., SSA has been proved to have better search accuracy, faster convergence rate, and more stable [27,28]. Therefore, this study proposed a traffic flow data repair method based on TGO-SSA-FCM and compared the repair effects of three different modes: TR, SR, and AR.

## 3. Materials and Methods

The main method of data repair is to use the FCM algorithm to repair the missing traffic flow data, and the specific optimization scheme is as follows:

(1) the t-SNE algorithm was used to reduce the dimension of data in AR mode to verify the correlation between multi-dimensional data and ensure the quality of the data matrix in the FCM algorithm.
(2) TGO algorithm was adopted to select the initial clustering center for the FCM algorithm.
(3) Sparrow search algorithm was adopted to optimize m and K of FCM.

### 3.1. Three Data Repair Mode

(1) Time correlation repair mode (TR) means to combine ‘weekly correlation’ traffic flow data into a matrix for data repair. The ‘weekly correlation’ data refers to the traffic data of the target traffic sensor for 7 consecutive days in the same week. Some studies [29,30] showed that traffic flow on working and non-working days followed different rules. Therefore, ‘week correlation’ data in this paper refers to the traffic flow data of the same traffic sensor on consecutive 5 working days in the same week. The data format is shown in Table 1.

(2) Spatial correlation repair mode (SR) means that the data of the ‘adjacent’ traffic sensor are used to predict the missing value of the data of the target traffic sensor. The ‘adjacent’ includes lane adjacency and position adjacency. The study [14] showed that the lane flow distribution of most roads was uneven. Therefore, ‘adjacent’ in this paper refers to location adjacency. We selected the traffic flow data of adjacent traffic sensors in different sections of the same road to perform curve fitting with the data of the target traffic sensor, and calculated the missing value of the target traffic sensor according to the fitting results. The data format is similar to that of the TR mode.
(3) Attribute value correlation repair mode (AR) replaces missing traffic values with other correct attribute values of the same traffic data. Studies [31,32] have shown a strong correlation between attribute values such as traffic flow, speed, and time occupancy, and the above attribute values could be used for data clustering analysis. This paper assumes that some traffic flow data of the infrared-based traffic sensor were wrong, which is regarded as missing data, and the speed and time share data are correct. The data format of AR mode is shown in Table 2.

### 3.2. Conventional Fuzzy C-Means Imputation Algorithm

FCM clustering algorithm is one of the most effective methods to deal with clustering problems in data mining and pattern recognition [33]. This method divides the data into different categories by maximizing the similarity between the data. The missing data can be calculated quantitatively according to the membership degree of the missing data to each category data clustering center. Assuming that the data is *X*, which contains *n* different elements, and each element contains *S* attributes, then *X* can be represented in the form of a matrix, as shown in Equation (1).

(1) $$X=\left(\begin{array}{ccc} x_{11} & \ldots & x_{1S}\\ \vdots & \ddots & \vdots \\ x_{n1} & \ldots & x_{nS} \end{array}\right)$$

where *n* is the number of samples obtained during data collection. In TR mode, it represents the traffic flow data collected at the *i*-th data collection interval on the *j*-th working day in a week.

In the FCM algorithm, the whole data set *X* has been divided into *K* different categories, where $c_{kj}$ represents the value of the *k*-th ($k=\left[1,2,\ldots ,K\right]$) clustering center in the *j*-th dimension. The steps of the traditional FCM algorithm are as follows:

Step 1: set parameter fuzzy weighting index *m* and classification quantity *K*, randomly select *K* clustering centers, and the clustering center dimension is the same as *X* dimension.

Step 2: due to the incomplete data, it is necessary to modify the distance equation from each data point to the cluster center in the traditional FCM. The local distance strategy of FCM in reference [34] is used to calculate the distance, as shown in Equation (2).

(2) $$D_{ik}=\sqrt{\frac{S}{\sum_{j=1}^{S}I_{ij}}\sum_{j=1}^{S}{\left\|x_{ij}-c_{kj}\right\|}^{2}I_{ij}}$$

where $D_{ik}$ represents the distance from the *i*-th data point to the *k*-th cluster center, S represents the data dimension. $I_{ij}$ indicates whether the *j*-th attribute value of the *i*-th data is missing, and its calculation equation is shown in Equation (3). $X_{M}$ represents missing data, $X_{P}$ represents non-missing data.

(3) $$I_{ij}=\left\{\begin{array}{c} 0,x\in X_{M}\\ 1,x\in X_{p} \end{array}\right.$$

Step 3: calculate the membership degree of each data point to the cluster center according to Equation (4).

(4) $$\mu_{ik}={\left({\sum_{t=1}^{K}\left(\frac{D_{ik}^{2}}{D_{it}^{2}}\right)}^{\frac{1}{m-1}}\right)}^{-1}$$

where $\mu_{ik}$ indicates the membership degree of the *I* data to the *k* cluster center, and *m* is the fuzzy weighted index.

Step 4: define the objective function of FCM as shown in Equation (5).

(5) $$J=\sum_{j=1}^{K}\sum_{i=1}^{n}\mu_{ij}^{m}D_{ij}$$

Step 5: judge whether the objective function satisfies the termination condition, and if so, end the iteration; otherwise, update the clustering center according to Equation (6), where $c_{k}$ represents the clustering center of the *k*-th cluster. Then return to step 2 until the minimum objective function value is obtained, and the optimal clustering center *C* and membership degree *U* are obtained at the same time.

(6) $$c_{k}=\frac{\sum_{i=1}^{n}\mu_{ik}^{m}x_{i}}{\sum_{i=1}^{n}\mu_{ik}^{m}}$$

Step 6: calculate the missing attribute value ${\bar{x}}_{ij}$ according to Equation (7).

(7) $${\bar{x}}_{ij}=\sum_{k=1}^{K}\mu_{ij}c_{kj}$$

### 3.3. Twice Grid Optimization Algorithm

The basic assumption of TGO is that the density of clustering centers of data is generally relatively large, and the distance between different clustering centers is relatively long. The basic idea is to study the distribution of the original data. Initially, the data is divided into uniform grids in terms of dimensions; then the grid is screened twice by density threshold and dimension threshold. Then the center point of the denser grid is taken as the alternative clustering center, and the Depth-First Search Strategy (DFSS) [35] is used to traverse the selected sets. Finally, the clustering center with the largest Xie-Beni index (XB) [36] is selected as the initial clustering center of the clustering algorithm.

Assume that the dataset is $X=\left\{x_{1},\ldots ,x_{j},\ldots ,x_{k}\right\}$, there are *k* dimensions in total, and the cluster number is *S*, which means the data is divided into *S* categories. Divide each dimension data into *m* grids according to the length of each dimension data, and each grid is marked as $Q_{jn}$, where $n=\left[1,2,\ldots ,m\right]$.

**Definition** **1.** *Grid length* $L_{j}$ *. It represents the range of each* $Q_{jn}$ *in the j-th dimension, and is calculated according to Equation (8).*

(8) $$L_{j}=\frac{\max\left\{x_{j}\right\}-\min\left\{x_{j}\right\}}{m}$$

**Definition** **2.** *Grid relative density* $q_{jn}$ *. It compares the relative density of grids in the same dimension, directly related to the number of data points in the grid, and is calculated according to Equation (9).* $q_{jn}$ *represents the relative density of the nth grid in the jth dimension of the data. With a larger value of* $q_{jn}$ *indicates the data points in the grid are denser, which means the possibility of becoming the cluster center is greater.*

(9) $$q_{jn}=\frac{g_{j1}+g_{j2}+\ldots +g_{j\min}}{L_{j}}$$

*where* $g_{ji}$ *is the i-th largest data point in grid* $Q_{jn}$ *, and* $g_{j\min}$ *is the smallest data point.*

**Definition** **3.** *Grid density ratio* $p_{jn}$ *. It represents the ratio of the relative density of grid a in the jth dimension, calculated according to Equation (10).*

(10) $$p_{jn}=\frac{q_{jn}}{\sum_{t=1}^{m}q_{jt}}$$

**Definition** **4.** *The center point of the grid*$C_{jn}$*. The data in the grid is represented by a central point, and the value is the median of the data in the grid*$Q_{jn}$.

**Definition** **5.** *The Depth-First Search Strategy (DFSS). We use the depth-first search algorithm in graph theory to traverse each grid center point according to the direction of dimension, and select one grid center point from each dimension in each search. a K-dimensional data as a cluster center point is composed.*

**Definition** **6.** *The High Similarity Data (HSD). It indicates that the data structure of the input data in each dimension is highly similar. The* $L_{j}$ *of each dimension data is similar, and the correlation coefficient of each dimension data is high.*

The steps of the TOG algorithm are as follows:

Step 1: determine the number of grids *m*, and calculate $L_{j}$, $q_{jn}$ and $p_{jn}$ respectively according to the Equations (8)–(10).

Step 2: the first time to optimize the grids. The density threshold ε to eliminate the grids where $p_{jn}$ is set below ε. Reduce the complexity of subsequent computation. For the remaining grids, the number of remaining grids in the *j*-th dimension are set as $R_{j}$. Calculate the grid center point $C_{j,r}$ according to Equation (11), where $r=\left[1,2,\ldots ,R_{j}\right]$.

(11) $$C_{j,r}=\left\{\begin{array}{cc} g_{(r+1)/2}\;,\; & r\;is\;odd\\ \frac{g_{r/2}+g_{r/2+1}}{2}\;,\; & r\;is\;an\;even\;number \end{array}\right.$$

The set composed of these grid center points is called the Primary clustering center, where $C_{F}=\left\{C_{1,1},\ldots ,C_{1,R_{1}},C_{2,1},\ldots ,C_{2,R_{2}},\ldots ,C_{k,R_{k}}\right\}$.

Step 3: combine the grid center points in $C_{F}$ according to DFSS to form multiple groups of clustering centers $C_{DFSS}=\left[C_{1},\ldots ,C_{i},\ldots ,C_{n}\right]$, where $C_{i}$ represents a group of cluster centers, and the dimension is S×k. $C_{i}$ consists of *S* cluster center points, and each cluster center point consists of *k* grid center points, which can be expressed as: $C_{i}=\left\{\left(c_{11},\ldots ,c_{1j},\ldots ,c_{1k}),\ldots ,(c_{S1},\ldots ,c_{Sj},\ldots c_{Sk}\right)\right\}$. DFSS can form multiple groups of cluster centers, and *n* is the number of groups.

Step 4: the second grid optimization. The purpose of optimization is to further reduce the computational complexity. The specific method is to set the dimension threshold (DT) and the cluster center threshold (CCT) according to the following two principles, and eliminate multiple groups of cluster centers that do not meet the requirements:

**Principle** **1.** *In* $C_{i}$ *, the distance of different clustering centers in the same dimension should be relatively far away.*

For principle 1, set the dimension threshold $DT=\left[DT_{1},\ldots ,DT_{j},\ldots ,DT_{k}\right]$, where $DT_{j}=\alpha L_{j}$, which represents the difference of each cluster center in the *j*-th dimension, and α is the dimension adjustment coefficient, the range of values is $\left[1.8,\;2\right]$. If $\left|c_{aj}-c_{bj}\right|\geq DT_{j}$ in $C_{i}$, where $\underset{a\neq b}{a,b}\in [1,2,\ldots ,S]$, j∈[1,2,…,k], keep the group of cluster centers $C_{i}$, otherwise remove it.

**Principle** **2.** *For HSD, we should ensure that the numerical gap of each dimension in* $C_{i}$ *is small.*

For principle 2, taking the TR pattern data in this paper as an example, each $L_{j}$ is close, and the average $L=\bar{L_{j}}$ can be taken as the length of the grid. In reality, when the data values of different dimensions of each cluster center of HSD are close, a better clustering effect can often be achieved (This will be demonstrated in the EXPERIMENTS Section). Set the cluster center threshold CCT=βL, which indicates the difference of the same cluster center in different dimensions, β is the adjustment coefficient of the cluster center, and the value range is $\left[0.8,\;1\right]$. If $\left|c_{sc}-c_{sd}\right|\geq CCT$ in $C_{i}$, where $\underset{c\neq d}{c,d}\in [1,2,\ldots ,k]$, s∈[1,2,…,S], keep the group of cluster centers $C_{i}$, otherwise remove it.

According to the above principles, the cluster centers in A are screened to obtain the set of multi-group cluster centers that meet the conditions, which is called the second clustering center $C_{S}$.

Step 5: determine the values of α and β in Step 4 according to the scale of $C_{S}$. The values are based on lower computational complexity and better clustering effect, which means calculating the *XB* index for each group of clustering centers in *n* according to Equation (12). A smaller *XB* index indicates a better clustering effect of the current cluster center. The cluster center with the smallest *XB* index in *m* is selected as the initial cluster center of FCM.

(12) $$XB=\frac{\sum_{i=1}^{n}\sum_{j=1}^{s}\mu_{ij}^{m}d\left(x_{i},c_{j}\right)}{n\times \underset{i\neq j}{\min}\;d\left(x_{i},c_{j}\right)}$$

where $\mu_{ij}^{m}$ represents the membership degree of sample $x_{i}$ to cluster center $c_{j}$, *m* is a fuzzy weighted index, and *d* represents the Euclidean distance.

### 3.4. Sparrow Search Algorithm

Sparrow Search Algorithm (SSA) is a new swarm intelligence optimization algorithm proposed by Xue [27] in 2020. Compared with traditional algorithms, SSA has strong optimization ability, fast convergence speed, and strong robustness.

SSA imitates the foraging process of sparrows, assuming that there are N sparrows in the D-dimensional space; the position of the *i*-th sparrow in the D-dimensional space is $x_{i}=\left\{x_{i1},\ldots ,x_{id},\ldots ,x_{iD}\right\}$, where $x_{id}$ represents the position of the ith sparrow in the *d*-th dimension.

All sparrows are divided into producers, scroungers, and scouts, and better food represents better adaptation.

**Definition** **7.** *Producers. It refers to the sparrows with high fitness in the population (the number is about 10–30% of the population). Their main function is to conduct extensive searches under the conditions of the population environment safety and obtain high-quality food information to guide the population to find higher fitness values. The location of the producers is updated as shown in Equation (13).*

(13) $$x_{id}^{t+1}=\left\{\begin{array}{cc} x_{id}^{t}\;\cdot \;\exp\left(\frac{-i}{\alpha \;\cdot \;T}\right) & R_{2}<S_{T}\\ x_{id}^{t}+Q\;\cdot \;L & R_{2}\geq S_{T} \end{array}\right.$$

*where* $x_{id}^{t}$*represents the position of the i-th sparrow in the d-th dimension,*α*is the random number between* $\left(0,1\right]$*, and T is the maximum number of iterations.* $R_{2}\in [0,1]$*and* $S_{T}\in [0.5,1]$*are divided into the early warning value and the safety value, respectively. Q is a random number with normal distribution. L is a matrix whose size is* 1×d*and elements are all 1.*

*When*$R_{2}$*is less than* $S_{T}$*, it indicates that the surrounding environment is safe, and the producers can search widely; otherwise, it indicates that the surrounding environment is dangerous and needs to move to other safe areas to search.*

**Definition** **8.** *Scroungers. It refers to the remaining sparrows other than the producers. Their main function is to follow the producers for local search. Once the producers find better food, the scroungers will move in the direction of the producers. The position update equation of the scroungers is shown in Equation (14)*

(14) $$x_{id}^{t+1}=\left\{\begin{array}{cc} Q\;\cdot \;\exp\left(\frac{xw_{d}^{t}-x_{id}^{t}}{i^{2}}\right) & \;i>\frac{n}{2}\\ xp_{d}^{t+1}+ & \\ \frac{1}{D}\sum_{d=1}^{D}\left(\left|x_{id}^{t}-xp_{d}^{t+1}\right|\;\cdot \;rand\left\{-1,1\right\}\right) & \;i\leq \frac{n}{2} \end{array}\right.$$

*where* $xw_{d}^{t}$*is the coordinate of the global worst position in the d-th dimension at the t-th iteration.* $xp_{d}^{t+1}\;$*represents the coordinates of the best location searched by the discoverer in the d-th dimension. When* $i>\frac{n}{2}$*, it indicates that the i-th participant does not get food and needs to fly to other areas; otherwise it means that a local search will be carried out near the optimal location*xp.

**Definition** **9.** *Scouts. A small number of sparrows are scouts (The number is about 10% of the population). The initial position of the scouts in the population is randomly generated. The main function of the scouts is to alert some scroungers when they do not have access to food in a small area. The position update equation of the scouts is shown in Equation (15).*

(15) $$x_{id}^{t+1}=\left\{\begin{array}{cc} xb_{d}^{t}+\beta \;\cdot \;\left|x_{id}^{t}-xb_{d}^{t+1}\right| & {\;f}_{i}\neq f_{g}\\ x_{id}^{t}+K\;\cdot \;\left(\frac{\left|x_{id}^{t}-xw_{d}^{t}\right|}{(f_{i}{-f}_{w})+\varepsilon }\right) & {\;f}_{i}={\;f}_{g} \end{array}\right.$$

*where* $xb_{d}^{t+1}$ *represents the coordinates of the optimal global position in the d-th dimensional space in the t + 1-th iteration, and* β *is a normal distribution random number with the mean value of 0 and the variance of 1.* $f_{g}$ *and* $f_{w}$ *are the global optimal adaptation value and the global worst adaptation value respectively, and K is a random number between* $\left[-1,1\right]$ *, which represents the direction of motion.* ε *is a minimal constant that prevents the denominator from being 0. When* $f_{i}\neq f_{g}$ *, it shows that the sparrow is at the edge of the population and is prone to danger. When* $f_{i}={\;f}_{g}$ *, it indicates that the sparrow is located in the middle of the population, which is necessary to approach other sparrows in time.*

The steps of the sparrow search algorithm are as follows: 
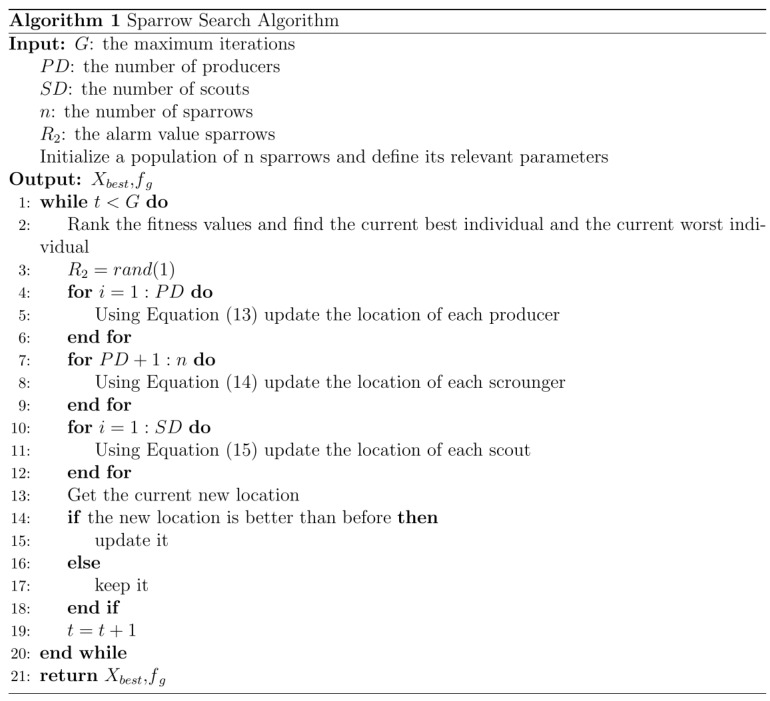


### 3.5. Data Repair Method Based on TGO-SSA-FCM Algorithm

TGO and SSA are used to jointly optimize the FCM algorithm for data repair. First, a complete data set is selected, and some of the data are randomly deleted. Then the predicted values of the missing data are obtained by using the TGO-SSA-FCM algorithm. Finally, the effectiveness of the repair method is verified by comparing the error of the missing value and the actual value. The data repair process based on the TGO-SSA-FCM algorithm is shown as follow:
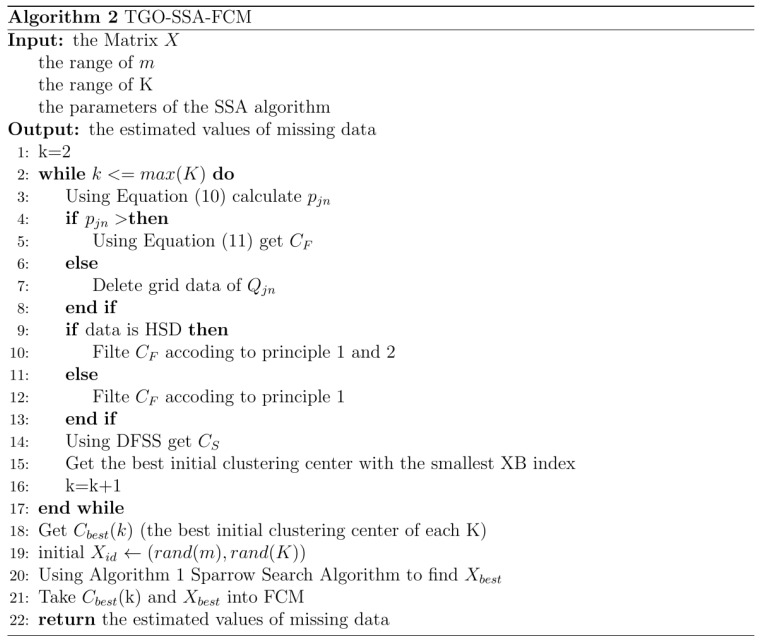


### 3.6. Evaluation Metrics

The root mean square error (RMSE) has been widely used to evaluate the deviation between the repaired value and the actual value, which can truly reflect the data repair effect. It is calculated according to Equation (16).

(16) $$\mathrm{RMSE}=\sqrt{\frac{1}{n}\sum_{i=1}^{n}{\left(y_{i}-{\hat{y}}_{i}\right)}^{2}}$$

where $y_{i}$ is the actual value, ${\hat{y}}_{i}$ is the predicted value, and *n* is the number of samples.

The relative accuracy (RA) [37] is an indicator of accuracy, which is used to describe the proportion of the number of relatively accurate data points to the total number of data points within a certain tolerance level, calculated according to Equation (17).

(17) $$\mathrm{RA}=\frac{n_{p}}{n}\times 100\%$$

where RA is the correlation coefficient, $n_{p}$ denotes the number of Percentage Absolute Error (PAE) in the ±10% error range, *n* denotes the data size.

## 4. Results

In this section, the measured data of traffic sensors were selected for the experiment. First of all, the correlation between the input data in the three repair modes was verified to ensure the quality of input data. Then we took two adjacent sensors as a group and selected three groups of sensor data for the experiment. Finally, six different algorithms were used to compare the repair effects.

### 4.1. Datasets

Three groups of the traffic sensor data were selected from three roads in Shunyi District, Beijing, China. Each group contained two sensors, the target sensor, and the adjacent sensor, separated by no more than two intersections. The data of the target sensor needed to be repaired and the data of the adjacent sensor was mainly used in SR mode. All the data came from the Information Center of the Beijing Municipal Commission of Transportation.

**Figure 1:**
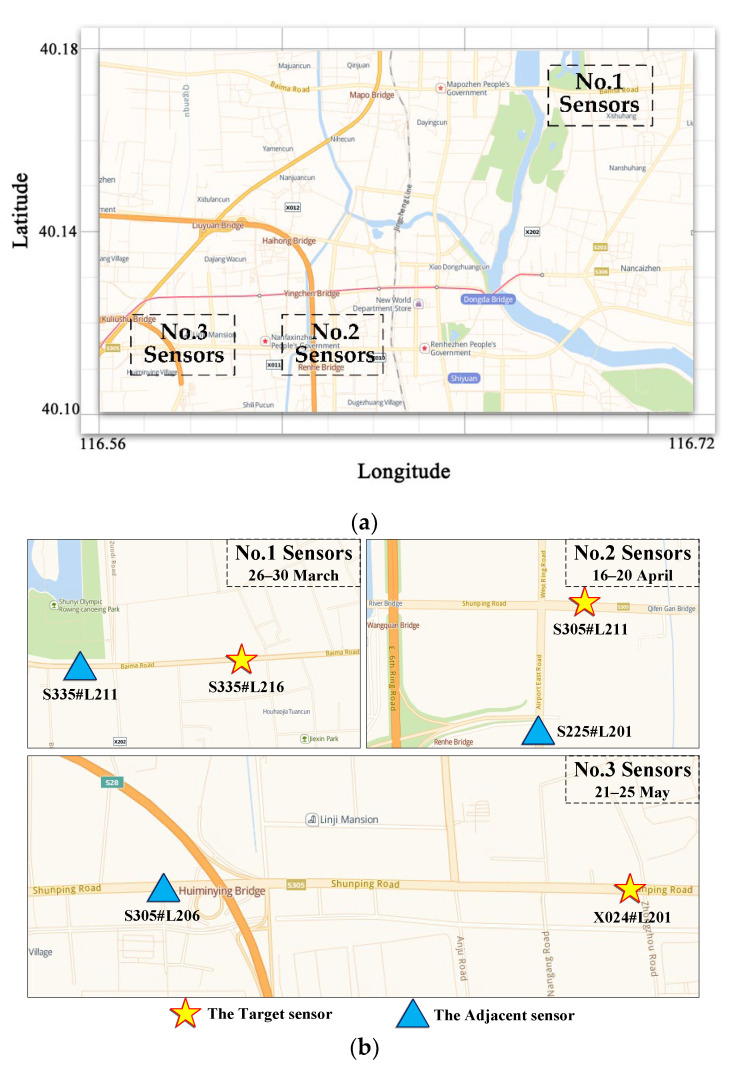
Basic information of 3 groups of sensors: (**a**) Latitude and longitude range of each group of sensors; (**b**) Specific position of each group of sensors.

We selected six months of data from 1 January 2018 to 1 July 2018 as the historical data. The three groups of sensors selected data from three different periods for experiments according to their own data integrity. The code names, location distribution, and data acquisition time of the three groups of sensors are shown in Figure 1. Holidays were avoided in each period, and the data was chosen for 5 consecutive working days. The data collection interval was 5 min, so the number of samples obtained by each sensor in each period was 1440, and the data were complete without missing data.

The group 1 and group 3 sensors worked as radio radars and transmitted data over wired and wireless networks, while the group 2 sensors collected data through sound waves and transmitted data over wired and wireless networks.

**Figure 2:**
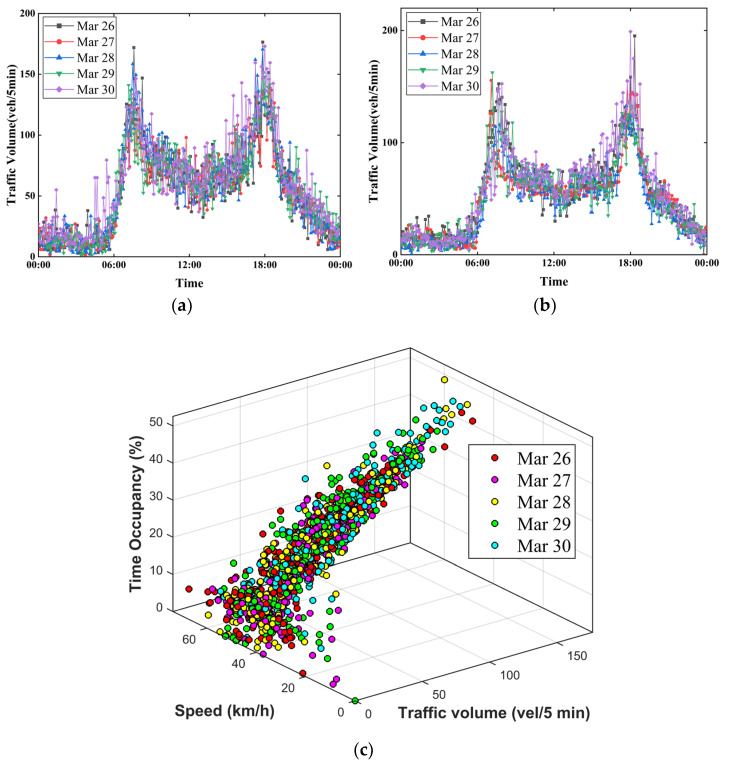
Input data for three modes. (**a**) Input data of TR mode; (**b**) Input data of SR mode; (**c**) Input data of AR mode.

The input of TR mode was the flow data of the target sensor, and the input of SR mode was the flow data of the target sensor and the adjacent sensor. Additionally, the input of AR mode was the flow data, speed data, and time occupancy data of the target sensor. Taking the data of sensor group 1 as an example, the input data of the three modes (TR, SR, AR) are shown in Figure 2a–c, respectively.

It can be seen in Figure 2 that there was an obvious ‘weekly correlation’ characteristic of traffic flow with the same sensor, and the traffic flow curve is basically the same for 5 consecutive working days. Compared with the two sensors, the variation trend of traffic flow is basically the same on the same time axis. From the attribute value of traffic data, when the traffic flow of target equipment is large, the time occupancy is relatively high, and the speed is relatively low. The above three attribute values conform to the basic law of traffic flow.

### 4.2. Spatial-Temporal Correlation Analysis for Traffic Data

Before setting up the FCM matrix, we need to verify the correlation of the data to ensure the quality of the input data, taking the data of the first group of sensors as an example.

**Figure 3:**
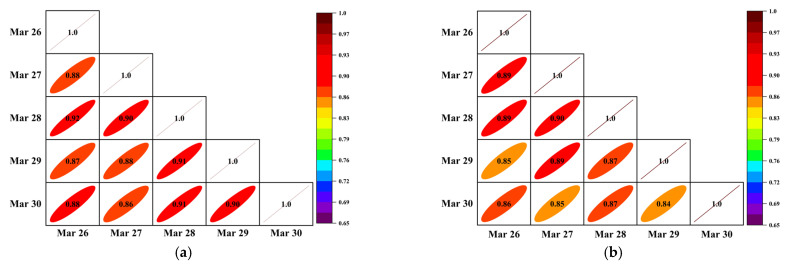
Pearson correlation coefficient of input Matrix. (**a**) Correlation matrix of TR mode; (**b**) Correlation matrix of SR mode.

For the one-dimensional flow data of TR and SR patterns, the Pearson correlation coefficient of each column of the matrix can be calculated directly. The correlation coefficient between the traffic flow on each working day can be obtained, shown in Figure 3a,b.

**Figure 4:**
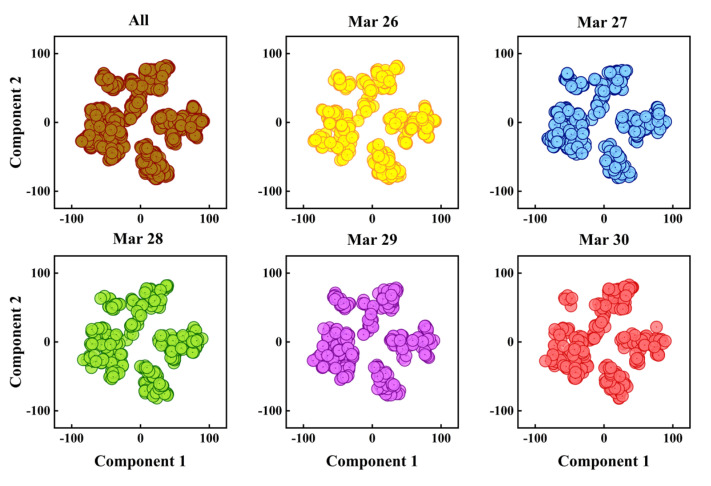
Visualization results of AR data with t-SNE.

It can be seen from Figure 4 that the correlation coefficient between the flow data of each day in the current period was more than 0.84, whether it is the target sensor or the adjacent sensor data. It can be there was a high correlation between the columns of the input data.

For the three-dimensional data of AR mode, the correlation coefficient could not be calculated directly. Consequently, the T-distributed Stochastic Neighbor Embedding (T-SNE) algorithm [38] was used to reduce the dimension. The three-dimensional data were projected onto the two-dimensional space composed of principal component 1 and principal component 2. The result is shown in Figure 4.

The six sub-graphs in Figure 4 represented the visualization results of the overall data and the data of each working day after dimensionality reduction. It could be seen that the overall data had been roughly divided into 3 parts, the data of 26 March–28 March is the same as the overall data in terms of data shape, and the data of 29 March and 30 March were divided into 4 parts, which were slightly different from the overall data.

To quantitatively describe the similarity of six groups of data, the baseline similarity proposed in reference [38] was used as the threshold, and the similarity between each day’s traffic volume and all traffic volume was calculated according to Equation (18).

(18) $$S_{i}=\frac{D_{B}-\left|D_{B}-D_{i}\right|}{D_{B}}$$

where $S_{i}$ represents the similarity between all traffic data and the traffic data of the *i*-th day, $D_{B}$ is baseline similarity, which represents the Spelman distance between all traffic data and randomly selected traffic data. The number of randomly selected data is 288 (the average number of traffic data on each working day). $D_{i}$ represents the Spelman distance between the traffic flow data of the *i*-th day and all traffic data. The calculated results are shown in Table 3.

From the statistical results, the similarity between each working day data and all data was above the baseline, indicating that the data structure was similar and could be summarized into a high-quality data matrix.

### 4.3. Preselection of Model Parameters

The initial clustering center of the traditional FCM algorithm was randomly selected, and the fuzzy weighting index m and classification number *k* were set manually. According to existing research [39,40], the range of *m* was $\left[1,2.4\right]$, and the range of *k* was $\left[2,\sqrt{n}\right]$. The value of parameter *n* was 288, which was the amount of sensor data in one day. In this paper, *n* was the amount of data of one day of traffic sensor 288, so the range of *k* was $\left[2,16\right]$.

**Figure 5:**
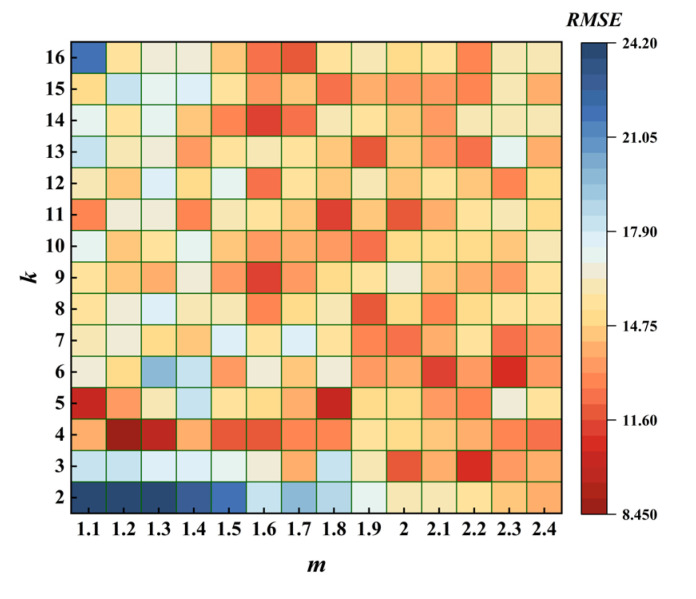
Traditional FCM parameter selection result.

Taking the data of the first sensor group as an example, input data of TR mode were selected to verify the above conclusion. We selected 72 samples (5% loss rate) randomly as the missing data. The traditional FCM algorithm was used to obtain the predicted value and calculate the RMSE between the predicted and the actual value. The calculation results are shown in Figure 5.

**Figure 6:**
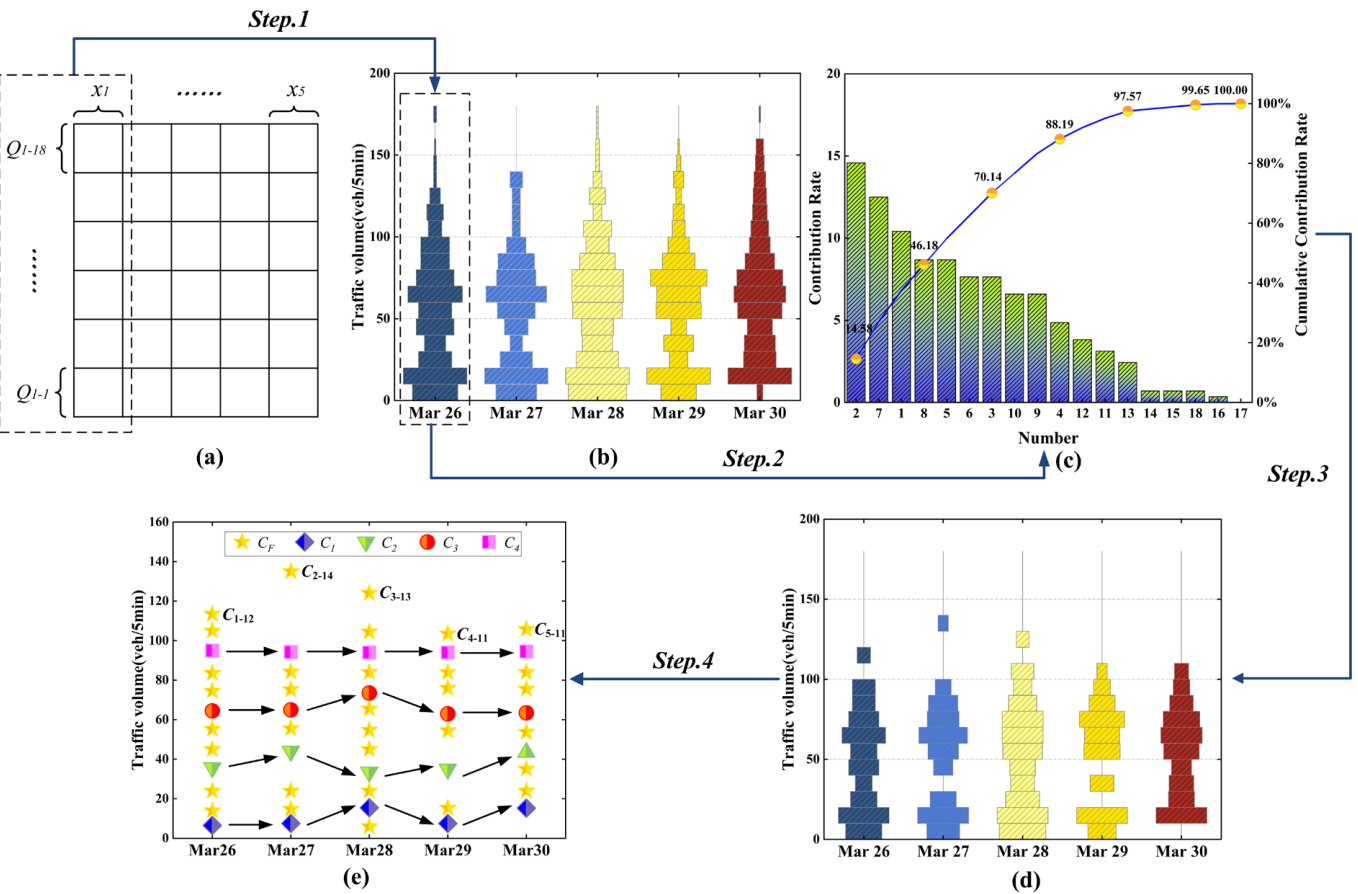
Example of TGO algorithm. (**a**) Data gridding; (**b**) Grid frequency distribution; (**c**) Cumulative contribution rate of each grid density; (**d**) Results of the first grid optimization; (**e**) Results of the second grid optimization.

As shown in Figure 6, the extreme range of RMSE is more than 15, indicating that the values of *m* and *k* have a great influence on the effect of data repair, and the repair effect was the best when *k* was 4 and *m* was 1.2.

To reasonably select the parameters of the FCM algorithm, the TGO algorithm was used to set the initial clustering center for FCM, and the sparrow search algorithm was used to select *m* and *K*.

### 4.4. Example of TGO Algorithm

Taking the TR pattern data of the first group of sensors as an example to show the process of optimizing the clustering center of FCM by using the TGO algorithm when *k* was 4. The data dimension was 5, and the value of m in TGO was set to 18, which indicated the data of each dimension had been divided into 18 grids of the same length. For example, the data of 26 March ($x_{1}$) was divided into $Q_{1–1}-Q_{1–18}$. Because the data belonged to HSD, the width of each grid was chosen as $\bar{L_{j}}=10$, as shown in Figure 6.

First of all, the original data in Figure 6a were divided into a grid of consistent size by column. Step 1 was to make statistics on the distribution interval of data in each column. For example, two dotted boxes were connected by Step 1, which indicated that the data of 26 March was transformed from the grid in Figure 6a into the frequency distribution diagram of each interval, as shown in Figure 6b. The width of each grid represented the frequency of the data points in the box. The more data points there were, the longer the grid’s width would be.

Step 2 calculated $p_{jn}$ of each grid. For example, Figure 6c sorted $p_{jn}$ of 26 March data in Figure 6b. The abscissa in the figure was the number of the grid on 26 March, and the two ordinates were the proportion and cumulative contribution rate, respectively.

Step 3 was the first time to optimize the grids. Set ε=3.5%, and the grid that $p_{jn}\leq 3.5$ was removed, and Figure 6b has been transformed into Figure 6d.

Step 4 calculated the $C_{jr}$ from the data of the remaining grid in Figure 6d to get $C_{F}$, which contained 53 grid center points. All $C_{jr}$ were the points in Figure 6e. Then the DFSS was used to traverse the points in $C_{F}$ to get several groups of cluster centers, in which a cluster center of a group of cluster centers could be expressed as $\left(c_{1–12},c_{2–14},c_{3–13},c_{4–11},c_{5–11}\right)$, where $c_{i-j}$ was the center point of the remaining grid $Q_{i-j}$. The cluster centers of each group were screened, and the $C_{S}$ in accordance with the condition was obtained.

**Figure 7:**
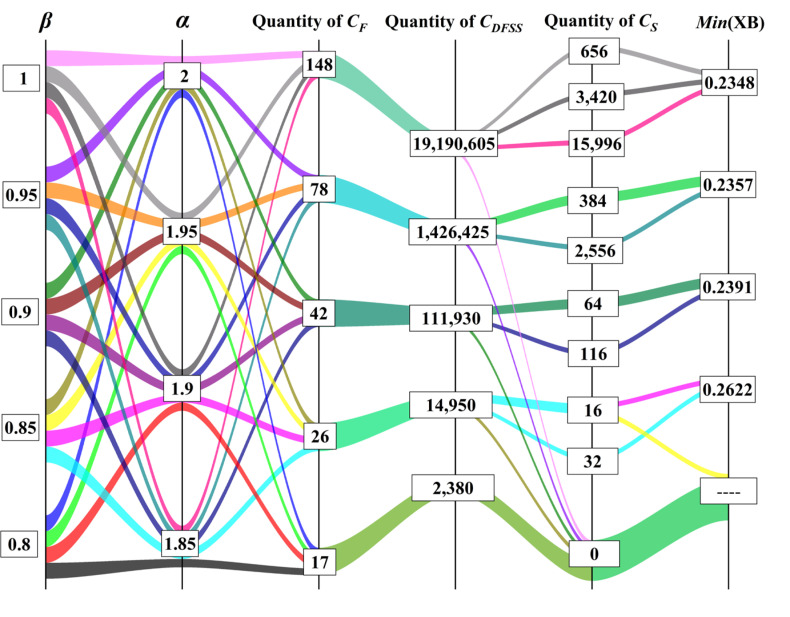
The selection result of thresholds.

In the calculation process, the values of α and β had a great influence on the complexity of the algorithm. Figure 7 was the computational complexity and clustering validity under different values.

As shown in Figure 7, the value of β directly affected the number of grid center points in $C_{F}$, and the scale of $C_{DFSS}$ increased greatly with the increase of β, while the value of α directly affects $C_{S}$. When the values of β and α were small, such as β=0.8, α=1.85, the size of $C_{DFSS}$ was 2380, which was easy to calculate. Nevertheless, $C_{S}$ was an empty set, and we could not get the clustering center that met the requirements. When the values of β and α were too large, such as β=1, α=2, the size of $C_{DFSS}$ was more than 19 million, which led to a great increase in the complexity of calculating the *XB* index, and the optimal *XB* index 0.2348 was very close to the *XB* index 0.2357 in other schemes.

**Figure 8:**
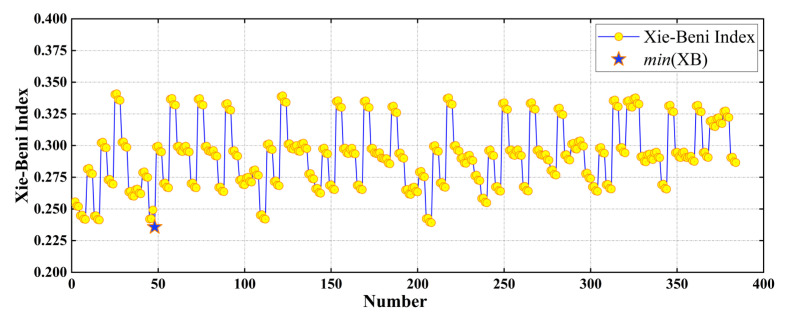
The XB index of each cluster center.

Finally, we weighed the computational complexity and clustering validity, set α=1.95, β=0.95, and got 384 groups of clustering centers. We calculated the *XB* index as shown in Figure 8.

When XB=0.2357, the corresponding clustering center was selected as the initial clustering center *C* of the FCM algorithm, which was shown as:

$$C=\left[\begin{array}{l} c_{1}\\ c_{2}\\ c_{3}\\ c_{4} \end{array}\right]=\left[\begin{array}{l} \left(6.5,7.5,15.5,7.5,15.25\right)\\ \left(35.75,44,33.5,35,43.5\right)\\ \left(64.5,65,73.5,63,63.5\right)\\ \left(95,94.25,94,94,94.5\right) \end{array}\right]$$

For easy understanding, we marked $c_{1}-c_{4}$ in Figure 6e, that is to say, the clustering effect was best when $c_{1}-c_{4}$ were taken as the initial clustering center.

### 4.5. Experimental Results

To verify the effectiveness of the improved algorithm and the repair effect of different repair modes, we selected 6 schemes for comparison, numbered S1–S6 respectively, and the details of each scheme are shown in Table 4.

Both S1 and S2 used the TGO-SSA-FCM algorithm to repair missing data. The difference was that the input of S1 was TR mode data, which belongs to HSD, so the grids were filtered according to principles 1 and 2. The input of S2 was AR mode data, which did not belong to HSD, so it used principle 1 only. The input of S3 was TR and SR mode data. The data of adjacent sensors were used to fit the target sensor data, and the missing data were estimated according to the fitted curve. The difference between S4 and S1 was that the TGO algorithm was not used. The difference between S5 and S4 was that another commonly used group optimization algorithm PSO optimized FCM as a contrast. S6 was a common historical mean algorithm for data repair. According to the detection time of different groups of detectors, the data mean value of missing data within one month at the same time was selected for data repair.

Where S3 used the method of curve fitting, and the data of the adjacent sensor were used to fit the data of the target sensor. Taking the data of the first group of sensors as an example, the optimal fitting results are shown in Table 5. The D1–D5 represented the data acquisition time (26–30 March), respectively, and “Model” represented the name of the model that worked best of all the curve models. “Curve fitting” referred to fitting the data of the target sensor with the data of the adjacent sensor on the same day. The $R^{2}$ were all above 0.78, which showed that the fitted trend line was close to the actual data curve.

The population size of the SSA and PSO algorithm in each scheme was set to N=30, the range of the maximum number of iterations was T=100, the accuracy of *m* was 0.01, and K was an integer. The number of the producers and scouts accounts were set for 20% and 10%, respectively, and $S_{T}=0.8$ in SSA. In the PSO algorithm, $c_{1}=c_{2}=1.5$, and ω showed a linear decreasing trend, which was calculated according to Equation (19), where $\omega_{max}=0.9$, $\omega_{min}=0.4$, iter was the current iterations.

(19) $$\omega =\omega_{max}-\frac{\omega_{max}-\omega_{min}}{T}\times iter$$

The experiments were implemented on Windows 10 with Intel Core i5-9300HF CPU @ 2.60 GHz processor, and RAM is 8 G. The GPU version was NVIDIA GeForce GTX 1660 Ti.

Among the 6 schemes, S3 was realized by SPSS 19. S1, S2, S4, S5, and S6 were realized by Pycharm2020.2.3.

**Figure 9:**
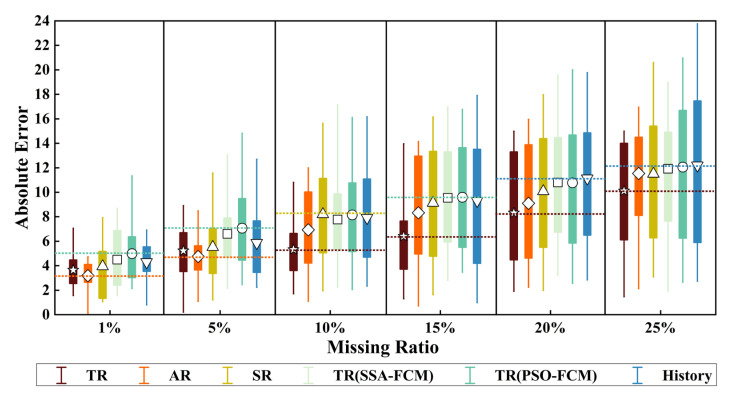
Absolute error of different schemes at different loss ratios.

We took the data from 26–30 March as an example. We compared the data repair effects of six schemes under different missing ratios, and the result is shown in Figure 9.

The mean of the absolute error is the most important information. A small mean value means there are more repaired data close to the real data. We added two “shot dot” lines to each missing ratio, representing the extreme value of the Mean Absolute Error (MAE) of each scheme. This makes it easier to show the difference between the mean and the extreme value of each alternative.

As shown in Figure 9, when the missing rate was 1%, S2 had the best repair effect with the absolute minimum error close to 0, and the MAE was the lowest among all schemes. S1 mode was also a better solution, superior to S3 and S6. Compared with S4, the MAE of S5 was higher and less stable. Although the distribution interval was smaller, the maximum absolute error of S5 was significantly higher than that of other schemes, which belonged to the poor scheme.

When the missing rate was 5%, the order of repair effect of each scheme was basically unchanged. S2 still had a small lead over S1, but on the whole, they both were still better schemes. S3 model and S6 still belonged to the medium scheme, but the maximum absolute error of S6 was larger.

When the missing rate increased from 10% to 25%, the advantages of the S1 model gradually emerged. Its MAE was the lowest among all schemes and gradually widened the gap with S2. Starting from the 10% error rate, the advantages of S3 and S6 gradually disappeared, and even the MAE of S3 was the highest at the missing rate of 10%. In addition, S4 was still slightly better than S5, but their repair effects were not ideal.

**Figure 10:**
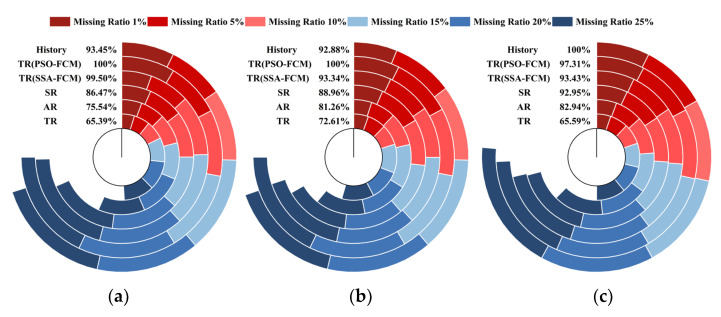
The RMSE of different schemes on different groups of sensor data. (**a**) RMSE of the first group of sensors; (**b**) RMSE of the second group of sensors; (**c**) RMSE of the third group of sensors.

To eliminate the chance of the experimental results, the data of the No.2 group of sensors (16–20 April) and the No.3 group of sensors (21–25 May) were selected to repeat the experiment, and the RMSE of the repaired value and the actual value was calculated. The experimental results are shown in Figure 10. Figure 10a–c represented the experimental results of group 1–group 3 sensors, respectively.

The 6 arcs in each subgraph represented 6 different schemes. Different colors represented RMSE values under different missing rates. Percentages in the figure represent the ratio of RMSE accumulated by the current scheme to the maximum RMSE scheme.

It could be seen from the figure that the repair effect of S1 was the best. Compared with the corresponding worst scheme, the cumulative RMSE ratio was only 60–70%. The repair effect of S2 was second. In the case of a low deletion rate (1% and 5%), the repair effect of S2 and TR was similar, and sometimes even better than that of S1. However, the repair of S2 was gradually inaccurate as the deletion rate increased. S3 could also perform some relatively accurate repairs, but the repair results for No.3 sensor data were not ideal. Overall, S4 was slightly better than S5, but the advantage was not large. S5 and S6 were relatively poor repair solutions.

**Figure 11:**
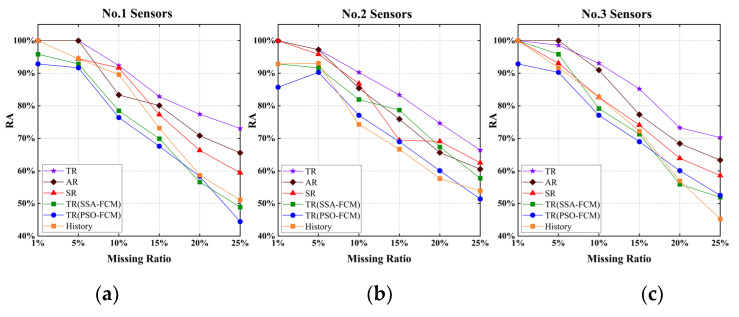
The RA of different schemes on different groups of sensor data. (**a**) RA of the No.1 sensors; (**b**) RA of the No.2 sensors; (**c**) RA of the No.3 sensors.

The Relative Accuracy (RA) of each scheme was calculated, and the results are shown in Figure 11. The above conclusion could be further verified under any missing rate condition; the RA of S1 ranks in the top 2. When the missing rate was low, S2 also performed well, but when the missing rate was greater than 10%, the performance of S2 would drop, but the repair was still better than most solutions. S3 fluctuated greatly under the data of different periods. When the March data was 10% missing rate, the RA was close to S1, but when the April data was 15% missing rate, the RA decreased significantly. The effect of S4–S6 was general, especially when the missing rate increased to 25%; about 50% of the data errors were above 10 veh/h.

**Figure 12:**
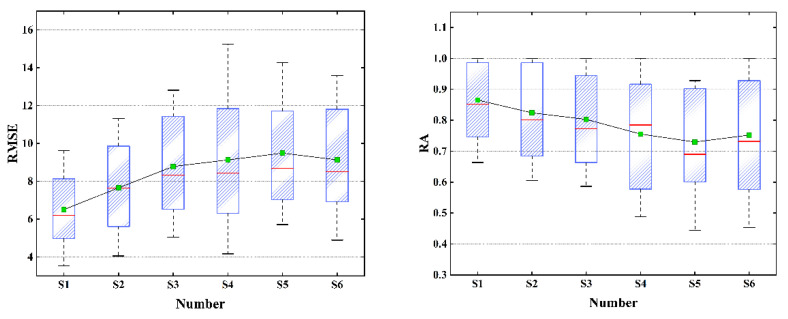
Comparison results of RMSE and RA.

Summarizing the data in Figure 10 and Figure 11, we get the box plot of each scheme, as shown in Figure 12. The red line in the figure was the median line, and the green square was the mean value. It could be seen that S1 had the best effect. The median and average values of RMSE and RA were significantly ahead of other schemes. S4 had the greatest volatility, but the overall effect was still ahead of S5. Both S5 and S6 were poor solutions.

### 4.6. Comparisons and Analyses of the Results

The reasons for the above experimental results were as follows:

(1) The repair effect of S1 was much better than that of S4 because the TGO algorithm was used to optimize the initial clustering center of FCM, and the clustering center of the FCM algorithm had a small XB index in the initialization stage, which ensured the accuracy of the clustering.
(2) S2 even achieved a better repair effect than S1 when the missing ratio was low. However, with the increase in the missing ratio, the repair effect was gradually surpassed by S1. The main reason was that there was no absolute correspondence between traffic flow, speed, and time occupancy in AR mode. For example, under normal circumstances, when the flow data was small the speed would be relatively high, and the time occupancy was low. However, although the flow data was small, the driver’s visual range was limited at night under the influence of lighting and other factors, which would cause a decrease in speed compared to the same flow during the day. Therefore, the inevitable volatility of the data itself would continue to accumulate with the increase of the missing rate, resulting in a decrease in the repair effect of S2 when the missing rate was high.
(3) The repair effect of S3 was relatively accurate, but the flow direction ratio of the traffic flow at the intersection was not fixed, which led to a decrease in the repair effect of S3. For example, for the third group of sensors, there were two intersections between the two sensors, and the repair effect of S3 was close to that of S4–S6. It could be concluded that the fitting of upstream and downstream sensor data has certain defects, and S3 would be feasible when the intersection flow direction ratio could be obtained stably.
(4) The effects of S4, S5, and S6 were similar. The effect of S4 was slightly better than that of S5, especially in the analysis of the second group of sensor data. Under the same initial clustering centers, the PSO algorithm fell more easily into local optimization than the SSA algorithm. S6 occasionally had unexpected effects in the case of a low miss ratio. However, due to the volatility of traffic flow data, the error of each time interval would accumulate, and led to a significant decrease in repair effect with the increase of loss ratio.

## 5. Conclusions and Future Work

Abundant traffic data collected by traffic sensors provide direct data support for formulating traffic management measures. Still, there is also a widespread data loss problem, making the repair method of traffic flow data attract much attention. To solve this problem, in addition to making use of the spatio-temporal characteristics of traffic flow, we should study the relationship between other attribute values of traffic data and traffic flow. Only by fully mining the internal relationship of data can the missing data be repaired with high quality.

The contribution of this paper is to establish a variety of data repair modes for finding the repair effects of different modes under different missing ratios. At the same time, the improved FCM algorithm has been used to cluster the traffic data, and the missing value was estimated by calculating the membership degree of each clustering center. To improve the algorithm, a TGO algorithm is proposed, which can provide a reliable initial clustering center for FCM, and combine with the SSA algorithm to solve the problem of FCM parameter selection.

The results showed that when the missing ratio is small, the AR mode often had a better repair effect, but there were many requirements for the input matrix. So it was more conventional to choose the TR mode for data repair, and with the increase in the deletion rate, the advantage of the TR mode was more significant, which has been verified in different groups of sensor data. The repair effect of the SR mode was unstable and was proportional to the correlation of the data between the two adjacent sensors. In terms of the data repair algorithm, the TGO-SSA-FCM algorithm was better than the traditional algorithm in data repair mean square error and relative accuracy and could reliably repair the missing data.

As for future work, we plan to select a denser road network and more sensors for testing and improvement. In addition, the relationship between other attributes of traffic data, such as headway and following percentage, may have the same potential, and how to repair the missing data with other attribute values is worth attention. Moreover, the parameter selection process of the TGO algorithm can be improved to reduce the computational complexity, which will lay a solid data foundation for the rapid analysis of historical traffic data and the formulation of future traffic control measures.

## Figures and Tables

**Table 1:** Data structure of TR mode.

Time Period	Monday	Tuesday	Wednesday	Thursday	Friday
00:00–00:05	19.50	20.50	14.00	16.50	23.00
00:05–00:10	16.00	?	17.00	15.00	19.50
23:50–23:55	12.00	?	15.50	?	20.00
23:55–00:00	20.00	12.50	?	27.00	23.50

The ‘?’ means the missing data.

**Table 2:** Data structure of AR mode.

Time Period	Flow (veh)	Speed (km/h)	Occupation (%)
00:00–00:05	19.50	53.06	7.69
00:05–00:10	?	63.70	4.71
23:50–23:55	11.5	42.51	4.87
23:55–00:00	?	47.94	6.08

The ‘?’ means the missing data.

**Table 3:** Similarity calculation result.

Parameters	26 March	27 March	28 March	29 March	30 March	Baseline
*D_i_*	0.8849	0.8740	0.8756	0.8807	0.8672	0.9302
*S_i_*	0.9513	0.9395	0.9413	0.9467	0.9323	

**Table 4:** The details of 6 schemes.

Number	Name	The Mode of Input Data	Method
S1	TR	TR mode data	TGO-SSA-FCM (Principle 1&2)
S2	AR	AR mode data	TGO-SSA-FCM (Principle 1)
S3	SR	SR & TR mode data	Curve fitting
S4	TR (SSA-FCM)	TR mode data	SSA-FCM
S5	TR (PSO-FCM)	TR mode data	PSO-FCM
S6	History	TR mode data	Historical mean

**Table 5:** The optimal curve fitting results.

Date	Model Summary					Parameters		
	Model	$R^{2}$	F	Sig.	Constant	b1	b2	b3
D1	Quadratic	0.813	619.943	0.000	−3.448	1.196	−0.002	
D2	Cubic	0.828	454.469	0.000	4.107	0.662	0.011	−6.947 × 10^−5^
D3	Linear	0.841	1510.432	0.000	3.534	1.108		
D4	Quadratic	0.810	608.382	0.000	−0.920	1.303	−0.003	
D5	Power	0.784	1038.502	0.000	2.525	0.790		

## Data Availability

The data presented in this study are available on request from the corresponding author. The data are not publicly available due to requirements of data provider.

## References

[1] Pavlyuk D. (2020). Temporal Aggregation Effects in Spatiotemporal Traffic Modelling. Sensors.

[2] Pozanco A., Fernández S., Borrajo D. Urban traffic control assisted by ai planning and relational learning. Proceedings of the 9th International Workshop on Agents in Traffic and Transportation.

[3] Castro-Neto M., Jeong Y., Jeong M., Han L.D. (2009). Online-SVR for short-term traffic flow prediction under typical and atypical traffic conditions. Expert. Syst. Appl..

[4] Cui Z., Henrickson K., Ke R., Wang Y. (2020). Traffic Graph Convolutional Recurrent Neural Network: A Deep Learning Framework for Network-Scale Traffic Learning and Forecasting. IEEE Trans. Intell. Transp. Syst..

[5] Sun B., Sun T., Jiao P. (2021). Spatio-Temporal Segmented Traffic Flow Prediction with ANPRS Data Based on Improved XGBoost. J. Adv. Transp..

[6] Lai X.C., Zhang L.Y. (2020). An overview of missing value filling methods. Theory and Method of Data Missing Value Filling Based on Machine Learning.

[7] Chan R.K.C., Lim J.M., Parthiban R. (2021). A neural network approach for traffic prediction and routing with missing data imputation for intelligent transportation system. Expert Syst. Appl..

[8] Zambrano-Martinez J., Calafate C., Soler D., Cano J., Manzoni P. (2018). Modeling and Characterization of Traffic Flows in Urban Environments. Sensors.

[9] Chen H., Grant-Muller S., Mussone L., Montgomery F. (2001). A Study of Hybrid Neural Network Approaches and the Effects of Missing Data on Traffic Forecasting. Neural Comput. Appl..

[10] Zhong M., Lingras P., Sharma S. (2004). Estimation of missing traffic counts using factor, genetic, neural, and regression techniques. Transp. Res. Part C Emerg. Technol..

[11] Chiou J., Zhang Y., Chen W., Chang C. (2014). A functional data approach to missing value imputation and outlier detection for traffic flow data. Transp. B Transp. Dyn..

[12] Qu L., Hu J., Li L., Zhang Y. (2009). PPCA-Based Missing Data Imputation for Traffic Flow Volume: A Systematical Approach. IEEE Trans. Intell. Transp. Syst..

[13] Tang J., Zhang G., Wang Y., Wang H., Liu F. (2015). A hybrid approach to integrate fuzzy C-means based imputation method with genetic algorithm for missing traffic volume data estimation. Transp. Res. Part C Emerg. Technol..

[14] Shang Q., Yang Z., Gao S., Tan D. (2018). An Imputation Method for Missing Traffic Data Based on FCM Optimized by PSO-SVR. J. Adv. Transp..

[15] Cheng Z., Wang W., Lu J., Xing X. (2020). Classifying the traffic state of urban expressways: A machine-learning approach. Transp. Res. Part A Policy Pract..

[16] Huang J., Mao B., Bai Y., Zhang T., Miao C. (2020). An Integrated Fuzzy C-Means Method for Missing Data Imputation Using Taxi GPS Data. Sensors.

[17] Luo X., Meng X., Gan W., Chen Y. (2019). Traffic Data Imputation Algorithm Based on Improved Low-Rank Matrix Decomposition. J. Sens..

[18] Han Y., He Z. (2020). Simultaneous Incomplete Traffic Data Imputation and Similarity Pattern Discovery with Bayesian Nonparametric Tensor Decomposition. J. Adv. Transport.

[19] Henrickson K., Zou Y., Wang Y. (2015). Flexible and Robust Method for Missing Loop Detector Data Imputation. Transp. Res. Rec. J. Transp. Res. Board.

[20] Wang S., Mao G. (2019). Missing Data Estimation for Traffic Volume by Searching an Optimum Closed Cut in Urban Networks. IEEE Trans. Intell. Transp. Syst..

[21] Zhang T., Zhang D., Gao J., Chen J., Jiang K. (2020). A novel approach of tensor-based data missing estimation for Internet of Vehicles. Int. J. Commun. Syst..

[22] Chen X., Wei Z., Li Z., Liang J., Cai Y., Zhang B. (2017). Ensemble correlation-based low-rank matrix completion with applications to traffic data imputation. Knowl.-Based Syst..

[23] Kazemi A., Meidani H. (2021). IGANI: Iterative Generative Adversarial Networks for Imputation With Application to Traffic Data. IEEE Access.

[24] Hathaway R.J., Bezdek J.C. (2002). Clustering incomplete relational data using the non-Euclidean relational fuzzy c-means algorithm. Pattern Recognit. Lett..

[25] Tian L., Jiang J., Tian L. (2019). Safety analysis of traffic flow characteristics of highway tunnel based on artificial intelligence flow net algorithm. Clust. Comput..

[26] Ming L.K., Kiong L.C., Soong L.W. (2011). Autonomous and deterministic supervised fuzzy clustering with data imputation capabilities. Appl. Soft. Comput..

[27] Xue J., Shen B. (2020). A novel swarm intelligence optimization approach: Sparrow search algorithm. Syst. Sci. Control. Eng..

[28] Li Y., Wang S., Chen Q. (2020). Comparative study of several new swarm intelligence optimization algorithms. Comput. Eng. Appl..

[29] Jia X., Dong X., Chen M., Yu X. (2021). Missing data imputation for traffic congestion data based on joint matrix factorization. Knowl-Based Syst.

[30] Su F., Dong H., Jia L., Sun X. (2017). On urban road traffic state evaluation index system and method. Mod. Phys. Lett. B.

[31] Xia J., Huang W., Guo J. (2012). A clustering approach to online freeway traffic state identification using ITS data. KSCE J. Civ. Eng..

[32] Zhao J., Liu P., Xu C., Bao J. (2021). Understand the impact of traffic states on crash risk in the vicinities of Type A weaving segments: A deep learning approach. Accid. Anal. Prev..

[33] Liang H., Chen H., Guo J., Jiang Y. (2021). An Evaluation Model of the Stuck Risks Based on Remote Sensor Network and Fuzzy Logic. IEEE Sens. J..

[34] Hathaway R.J., Bezdek J.C. (2001). Fuzzy c-means clustering of incomplete data. IEEE Trans. Syst. Man Cybern. Part B Cybern..

[35] Cui L., Li G., Lin Q., Du Z., Gao W., Chen J., Lu N. (2016). A novel artificial bee colony algorithm with depth-first search framework and elite-guided search equation. Inform. Sci..

[36] Xie L., Beni G. (1991). A vilidity measure for fuzzy clustering. IEEE Trans. Pattern Anal. Mach. Intell..

[37] Aydilek I.B., Arslan A. (2013). A hybrid method for imputation of missing values using optimized fuzzy c-means with support vector regression and a genetic algorithm. Inform. Sci..

[38] Yi D., Su J., Liu C., Quddus M., Chen W. (2019). A machine learning based personalized system for driving state recognition. Transp. Res. Part C Emerg. Technol..

[39] Ozkan I., Türkşen I.B. (2012). MiniMax *ε*-stable cluster validity index for Type-2 fuzziness. Inform. Sci..

[40] Zahid N., Abouelala O. (1999). Unsupervised fuzzy clustering. Pattern Recognit. Lett..

